# Distal triceps tendon rupture repair results in high return to sport rates for amateur and professional athletes: a systematic review

**DOI:** 10.1016/j.xrrt.2026.100694

**Published:** 2026-02-06

**Authors:** Caleb J. Vandenberg, Daniel C. Touhey, Robert H. Brophy, Matthew V. Smith, Derrick M. Knapik

**Affiliations:** Department of Orthopaedic Surgery, Washington University School of Medicine, St. Louis, MO, USA

**Keywords:** Distal triceps tendon rupture, Surgical repair, Return to sport, Patient-reported outcomes, Complications, Systematic review

## Abstract

**Background:**

Distal triceps tendon ruptures (DTTRs) typically occur in active patients engaged in sports and exercises involving resisted elbow extension. Surgical repair is generally recommended to restore arm function and strength. There is currently limited evidence to guide expectations for return-to-sport (RTS) among active patients. The purpose of this investigation is to systematically review the literature to better understand outcomes after repair of DTTR, particularly in amateur and professional athletes, with a focus on RTS rate and timing, along with the incidence of post-operative complications.

**Methods:**

Studies included in PubMed, EMBASE, and Cochrane Library databases from inception to September 2025 reporting on patients identified as athletes undergoing DTTR repair were identified. Inclusion criteria included studies reporting injury mechanism, tear characteristics (extent and location), competition level, RTS rate and timing, and post-operative outcomes, including complications and/or reoperations, and patient-reported outcomes.

**Results:**

A total of 31 studies, consisting of 277 athletes (n = 283 elbows) undergoing DTTR repair, with a weighted mean follow-up of 23.4 (range, 12-108) months, were identified. The weighted mean patient age was 36.2 years (range, 12-62 years), with 96.4% of patients being male. Sports-related injuries were the most frequently reported etiology (59.0%; n = 131/222), with the dominant arm being affected in 55.6% (n = 50/90) of cases. Weightlifting (40.7%, n = 87/214) and American football (26.6%, n = 57/214) were the most commonly reported athletic activities. The weighted mean time interval from injury to repair was 2.3 months (range, 0.1-12). Repair techniques included open repair using transosseous bone tunnel (45.3%, n = 97/214) and primary suture (40.2%, n = 86/214) repair. Rerupture was the most frequently reported complication (4.4%; n = 9/206), with 7.8% (n = 16/206) undergoing reoperations, including revision repair in 2.4% (n = 5/206). A total of 93.0% (n = 252/271) of athletes reported successful RTS at a weighted mean of 5.2 (range, 1.6-10.6) months, with 63.6% (n = 42/66) returning to their prior or higher level of competition. The weighted mean post-operative visual analog scale score was 1.7 (n = 97), with Disabilities of the Arm, Shoulder, and Hand score of 4.1 (n = 24), Quick Disabilities of the Arm, Shoulder, and Hand score of 8.1 (n = 71), and Mayo Elbow Performance Score of 89.1 (n = 119).

**Conclusion:**

The vast majority of patients undergoing DTTR repair are male, most commonly engaged in weightlifting or American football. Successful RTS was reported in 93% of patients at a mean of 5.2 months following repair, while tendon rerupture was reported in 4.4% and reoperations in 7.8%.

## Introduction

Distal triceps tendon ruptures (DTTRs) account for less than 1% of all tendon injuries.^3^^,^^5^^,^^31^^,^^51^ DTTRs are most commonly reported in middle-aged males and are generally associated with forceful eccentric loading across the elbow, typically during activities such as weightlifting, football, or as a result of a traumatic fall.^26^^,^^50^ In athletes and other active individuals, DTTR results in disability due to loss of elbow extension strength, limiting performance and the potential to return to preinjury activity levels. While partial DTTR may be managed conservatively, the high functional demands of athletic patients typically necessitate repair for symptomatic ruptures to restore function and strength.^46^^,^^51^

Outcomes following DTTR repair are currently limited to small case series and retrospective cohort studies, often with heterogeneous patient populations and surgical techniques. Reported return-to-sport (RTS) rates following repair are generally favorable, with studies on professional football players and military service members observing successful RTS in greater than 90% of patients.^5^^,^^11^^,^^13^^,^^29^ However, considerable variability exists in the RTS timing, criteria defining successful RTS, as well as the level of competition. In addition, complications such as rerupture, persistent pain, and/or the presence of post-operative neurologic symptoms are inconsistently reported, further confounding comparisons across studies. As such, there remains a limited understanding of RTS outcomes in athletes following DTTR repair. The purpose of this study is to systematically review the current literature to better understand RTS outcomes, timing, and the incidence of complications among athletes undergoing DTTR repair.

## Methods

### Search strategy and eligibility criteria

A systematic review was conducted in accordance with the 2020 Preferred Reporting Items for Systematic Reviews and Meta-Analyses statement, utilizing a Preferred Reporting Items for Systematic Reviews and Meta-Analyses checklist.^37^ A literature search was conducted on September 25, 2025, to identify studies reporting on patients with DTTR undergoing repair. Two authors (C.J.V., D.C.T.) independently performed a qualitative systematic review of the literature using the PubMed, Cochrane Database for Systematic Reviews, Cochrane Central Register of Controlled Trials, and Embase databases from inception to September 2025. The search was performed using various combinations of the following search terms with Boolean operators: “distal triceps tendon,” “injury,” “rupture,” “tear,” “tendon repair,” “return to sport,” return to play, “return to activity,” and “athlete.”

Inclusion criteria consisted of clinical studies written in English reporting on patients identified as athletes with DTTR with reported mechanism of injury and sporting activity, pertinent physical examination findings (eg, range of motion, palpable tendon defect), rupture characteristics (eg, defect location [eg tendon avulsion, tear at tendinous insertion, midsubstance tear]), surgical technique (eg suture repair, suture anchor, transosseous tunnel vs. tendon–tendon vs. tendon–bone repair), graft augmentation, reported post-operative complications and reoperations, as well as the rate of successful RTS, including RTS timing and level of competition (amateur, collegiate, semiprofessional, professional). Exclusion criteria consisted of non-English studies; cadaveric, biomechanical, and animal studies; previous meta-analyses and systematic reviews; review articles; editorial commentaries; as well as studies consisting of patients not identified as athletes either by means of professional sport involvement or consistent engagement in sport at the amateur level.

Title and abstract screenings were independently performed by 2 authors (C.J.V., D.C.T.), followed by a full-text screening to determine which studies met inclusion criteria. The senior author (D.M.K.) was assigned to consult if any disagreements were encountered, classified as any discrepancy raised by one of the 2 authors for article inclusion, of which none were encountered. References from the included studies were reviewed to ensure that all studies meeting the inclusion criteria were identified and included.

### Data extraction

For studies meeting inclusion criteria, the following study characteristics from each article were recorded and entered into a Microsoft Excel spreadsheet (version 16.101, Redmond, WA): study title, year published, first author, level of evidence, patient demographics (mean age at time of surgery, sex), mechanism of injury, pertinent physical examination findings, mean interval time from injury to surgery, presence of concomitant injuries, tear characteristics (location, severity), surgical technique, graft type if reconstruction was performed, mean follow-up time, the incidence of post-operative complications, any patient-reported outcomes, ability to RTS, RTS time, and level of competition.

### Study quality assessment

To assess bias, a methodological quality assessment was performed by 2 independent authors (C.J.V., D.C.T.) using the Joanna Briggs Institute (JBI) critical appraisal tools for case series (Appendix Table 1) and the JBI critical appraisal tool for case reports (Appendix Table 2). A third author (D.M.K.) was consulted in case of any disagreements, of which none were encountered. The JBI critical appraisal tools consist of 10 questions for case series and 8 questions for case reports, with each question scored as follows: “Y,” yes; “N,” no; “U,” unclear; and “NA,” not applicable. The total percentage of “Y” (yes) responses was recorded for each study and each question, with the highest achievable score being 100% (range, 0%-100%).^4^^,^^32^

### Data analysis

Patient demographics and study characteristics were compiled and analyzed using Microsoft Excel (version 16.101, Redmond, WA). Variables such as patient age, body mass index, time from injury to surgery, and mean follow-up time were calculated and displayed as weighted means. Individual sporting activities were classified according to the relative injury risk categories defined by Rice.^40^ These categories ranged from greatest to least risk of injury and included contact and limited-contact sports. Contact sports included American football, basketball, jiu-jitsu, Kabbadi, snowboarding, and soccer; limited-contact sports consisted of weightlifting and bouldering.

## Results

**Figure 1:**
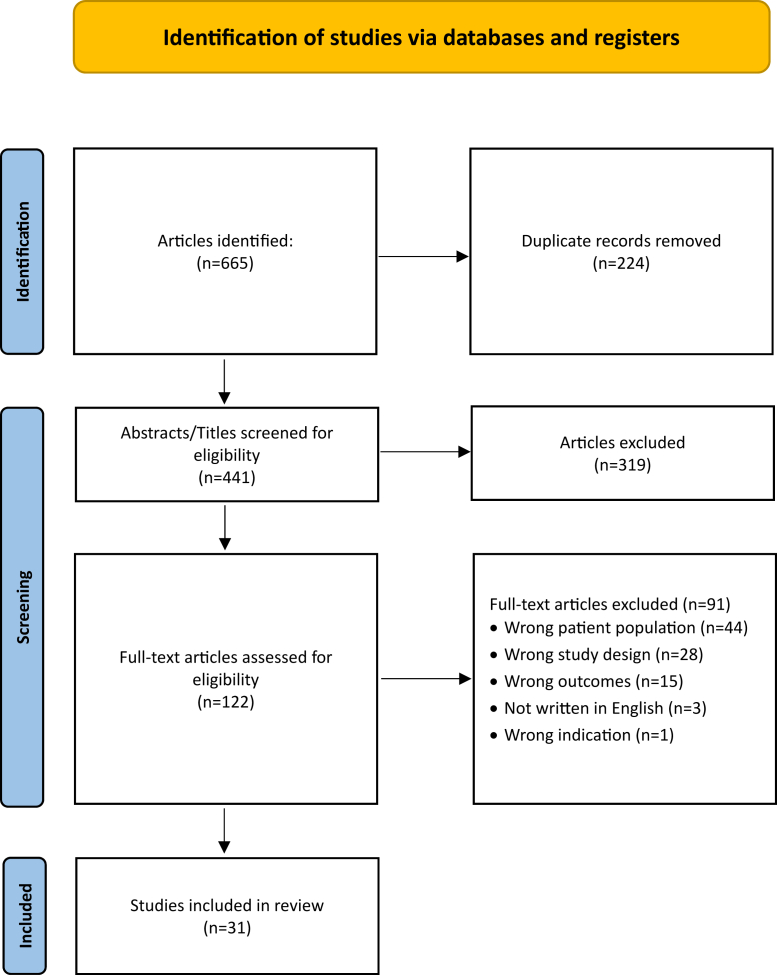
PRISMA diagram. *PRISMA*, Preferred Reporting Items for Systematic Review and Meta-analyses.

The initial literature search identified 665 articles. After removal of duplicates, 441 articles underwent title and abstract screening. A total of 122 studies were selected for full-text review. Thirty-one studies published between 1984 and 2025 were identified as meeting inclusion criteria (Fig. 1), encompassing 277 athletes with 283 elbows undergoing DTTR repair. A total of 22 studies were of Level V evidence,^7^^,^^10^^,^^12^^,^^14^^,^^15^^,^^17^^,^^19^^,^^20^^,^^24^^,^^30^^,^^33^, ^34^, ^35^, ^36^^,^^38^^,^^39^^,^^41^, ^42^, ^43^^,^^47^^,^^52^^,^^53^ while 9 were of Level IV evidence.^1^^,^^5^^,^^11^^,^^13^^,^^16^^,^^18^^,^^25^^,^^27^^,^^29^ The methodological quality of included studies was assessed using the JBI critical appraisal tools [Appendix Tables 1 and 2]. When assessing the mean “yes” score for case series was 87% (range, 70-100%), while the mean “yes” score for case reports was 89% (range, 63-100%).

### Study and patient characteristics

A total of 277 athletes undergoing surgical repair for DTTR were identified from the 31 included studies (Table I). The weighted mean patient age was 36.2 years (range, 12-62 years), with 96.4% (n = 267/277) of patients being male. Body mass index was reported for 38.6% (n = 107/277) of patients, with a weighted mean of 32.7 kg/m^2^. In the subset of patients where arm dominance was reported, the dominant extremity was involved in 55.6% (n = 50/90) of cases. Bilateral DTTR repairs were performed in 2.2% (n = 6/277) of patients. Professional athletes comprised 24.8% (n = 53/214) of patients,^12^^,^^13^^,^^17^^,^^29^^,^^35^^,^^36^^,^^42^ while the remainder were either amateur athletes or athletes of an unspecified competitive level. The weighted mean duration of follow-up was 23.4 months (range, 12-108 months), while follow-up was not reported in 4 studies.^1^^,^^12^^,^^13^^,^^19^

**Table I:** Overview of clinical studies and patients.

Study	LOE	No. of patients	% male patients	Mean age, yr	MOI	Sporting activity	Repair technique	Concomitant Procedure(s) performed	Complications/Reoperations	Outcomes (% RTS, other post-operative scores)	Mean follow-up, mo
Agarwalla^1^	IV	68	93	46.6	Traumatic (n = 44), sports-related (n = 22)	Running (n = 22), weightlifting (n = 9), swimming (n = 14), golf (n = 18), basketball (n = 14), cycling (13), baseball (n = 16), football (n = 7), and soccer (n = 3)	POSR (n = 25), OSA (n = 18), OTBT (n = 25)	None	None	RTS (89.7), VAS (2), QuickDASH (8.2), and MEPS (89.5)	NR
Balazs^5^	IV	54	100	37	NR	Weightlifting (n = 54)	POSR (n = 54), graft augmentation (n = 1)	Radial head fracture (n = 1), osteochondral injury of capitellum (n = 2), UCL injury (n = 5), anconeus tear (n = 3), flexor–pronator injury (n = 5), and biceps tendon tear (n = 1)	TRF (n = 6), prolonged weakness/pain (n = 5), nerve palsy (n = 1)	RTS (93.8)	18
Bunshah^7^	V	1	100	40	Traumatic (n = 1)	Weightlifting (n = 1)	POSR (n = 1)	None	None	RTS (100), MEPS (85)	12
Chorba^10^	V	1	100	35	Sports-related (n = 1)	Weightlifting (n = 1)	NR	None	None	RTS (100)	24
Dunn^11^	IV	37	97	38.4	Atraumatic (n = 3), Sports-related (n = 16), direct blow/fall (n = 8)	NR	OTBT (n = 9), TBR (n = 3)	UCL injury (n = 2), flexor–pronator mass injury (n = 2)	TRF (n = 1)	RTS (83.8), DASH (4.7), MEPS (85.4)	49.8
Figueiredo^12^	V	1	100	42	Direct blow/fall (n = 1)	Jiu-jitsu (n = 1)	OTBT (n = 1)	None	None	RTS (100)	NR
Finstein^13^	IV	37	100	nr	Sports-related (n = 37)	Football (n = 37)	NR	None	None	RTS (100)	NR
Goodrich^14^	V	1	100	37	Direct blow/fall (n = 1)	Running (n = 1), basketball (n = 1)	OTBT (n = 1)	None	None	RTS (100)	12
Greer^15^	V	1	100	43	Direct blow/fall (n = 1)	Weightlifting (n = 1)	OTBT (n = 1)	None	None	RTS (100)	6
Gruber^16^	IV	22	100	44.3	Traumatic (n = 2), atraumatic (n = 1), sports-related (n = 18), and direct blow/fall (n = 1)	NR	OTBT (n = 22)	None	None	RTS (95.5), VAS (1.5)	38
Gupta^17^	V	1	100	25	Sports-related (n = 1)	Kabaddi (n = 1)	OTBT (n = 1), graft augmentation (n = 1)	None	None	RTS (100), DASH (4.2)	60
Hall^18^	IV	7	100	38	Sports-related (n = 5), direct blow/fall (n = 2)	Golf (n = 1), lacrosse (n = 1), cycling (n = 1), rugby (n = 1), and hockey (n = 1)	OTBT (n = 7)	None	Infection-related partial rerupture (n = 1), intermittent ulnar neuropathy (n = 1)	RTS (100), VAS (0), DASH (1.3), MEPS (99.3)	49.2
Hernandez^19^	V	1	0	12	Direct blow/fall (n = 1)	Soccer (n = 1)	OSA (n = 1)	None	None	RTS (100)	NR
Holmes^20^	V	1	100	50	Direct blow/fall (n = 1)	Weightlifting (n = 1)	Graft augmentation (n = 1)	None	None	RTS (100)	18
Khalil^24^	V	1	100	13	Sports-related (n = 1)	Football (n = 1)	OSA (n = 1)	UCL rupture (n = 1)	None	RTS (100)	12
Kose^25^	IV	8	75	25.1	Traumatic (n = 1), sports-related (n = 3), and direct blow/fall (n = 4)	Weightlifting (n = 1)	OTBT (n = 8)	Radial head fracture (n = 2)	Ulnar nerve entrapment (n = 1), posterior interosseus nerve palsy (n = 1)	RTS (100)	18.8
Lempainen^27^	IV	10	100	35	Sports-related (n = 9), direct blow/fall (n = 1)	Weightlifting (n = 10)	POSR (n = 6), TBR (n = 4)	None	None	RTS (90)	60
Mair^29^	IV	11	100	29	Sports-related (n = 11)	Football (n = 11)	TBR (n = 11)	None	TRF (n = 1)	RTS (90.9)	36.0
Mangano^30^	V	1	100	52	Sports-related (n = 1)	Weightlifting (n = 1)	OTBT (n = 1)	None	None	RTS (100)	24
Naito^33^	V	1	100	18	Sports-related (n = 1)	Football (n = 1)	OTBT (n = 1)	None	None	RTS (100)	24
Naula^34^	V	1	100	52	Sports-related (n = 1)	Weightlifting (n = 1)	TBR (n = 1), arthroscopic (n = 1)	None	None	RTS (100), QuickDASH (18), MEPS (95)	7
Nikolaidou^35^	V	1	100	28	Sports-related (n = 1)	Weightlifting (n = 1)	TBR (n = 1), graft augmentation (n = 1)	None	None	RTS (100)	18
Ntourantonis^36^	V	1	100	32	Atraumatic (n = 1)	Weightlifting (n = 1)	TBR (n = 1)	None	None	RTS (100), MEPS (100), OES (48)	12
Pilih^38^	V	1	100	32	Direct blow/fall (n = 1)	Weightlifting (n = 1)	TBR (n = 1), graft augmentation (n = 1)	None	Repair failure (n = 1)	RTS (100), DASH (0), OES (44)	12
Qin^39^	V	1	0	27	Direct blow/fall (n = 1)	Climbing (n = 1)	TBR (n = 1)	None	None	RTS (100), DASH (18.3), MEPS (85), OES (42)	5.5
Schreiderer^41^	V	1	100	30	Direct blow/fall (n = 1)	Snowboarding (n = 1)	TBR (n = 1)	None	None	RTS (100)	3
Sherman^42^	V	1	100	34	Direct blow/fall (n = 1)	Weightlifting (n = 1)	TBR (n = 1)	None	None	RTS (100)	6
Shivdasani^43^	V	1	100	53	Sports-related (n = 1)	Weightlifting (n = 1)	TBR (n = 1)	None	None	RTS (100), MEPS (100)	12
Tramer^47^	V	1	100	38	Sports-related (n = 1)	Weightlifting (n = 1)	TBR (n = 1)	Biceps tendon repair (n = 1)	None	RTS (100)	12
Weistroffer^52^	V	1	100	49	Sports-related (n = 1)	Weightlifting (n = 1)	TBR (n = 1), graft augmentation (n = 1)	None	None	RTS (100)	24
Welborn^53^	IV	2	100	50	Sports-related (n = 1), direct blow/fall (n = 1)	Golf (n = 1), wrestling (n = 1)	TBR (n = 2)	None	None	RTS (100), QuickDASH (0), MEPS (100)	42

*DASH*, Disabilities of the Arm, Shoulder, and Hand; *LOE*, level of evidence; *MEPS*, Mayo Elbow Performance Score; *MOI*, mechanism of injury; *NR*, not reported; *OES*, Oxford Elbow Score; *OSA*, open suture anchor; *OTBT*, open transosseous bone tunnel; *POSR*, primary open suture repair; *TBR*, tendon–bone repair; *TRF*, traumatic repair failure; *RTS*, return to sport; *VAS*, visual analog scale; *UCL*, ulnar collateral ligament.

### Injury characteristics, associated injuries

Rupture characteristics were reported across 59.2% (n = 164/277) of patients, with complete tears^1^^,^^7^^,^^11^^,^^14^^,^^15^^,^^17^, ^18^, ^19^, ^20^^,^^25^^,^^27^^,^^29^^,^^30^^,^^33^, ^34^, ^35^, ^36^^,^^39^^,^^41^, ^42^, ^43^^,^^52^^,^^53^ being most frequent (58.5%, n = 96/164), and partial tears^1^^,^^10^, ^11^, ^12^^,^^18^^,^^24^^,^^27^^,^^38^^,^^47^ comprising 41.5% (n = 68/164) of injuries. Location of complete rupture was reported for 27.1% (n = 26/96) of patients, including tendon avulsions from the olecranon^14^^,^^15^^,^^19^^,^^25^^,^^27^^,^^30^^,^^33^, ^34^, ^35^, ^36^^,^^39^^,^^42^^,^^43^^,^^52^^,^^53^ in 96.2% (n = 25/26) and tears at the musculotendinous junction^17^ in 3.8% (n = 1/26). The location of partial rupture was reported in 14.7% (n = 10/68) of patients, with tendon avulsions and tears at the tendinous insertion^10^^,^^24^^,^^47^ accounting for 30.0% (n = 3/10) and tears at the musculotendinous junction^12^ accounting for 70% (n = 7/10). The mechanism of injury was reported in 80.1% (n = 222/277) of patients, with sports-related injuries^1^^,^^10^^,^^11^^,^^13^^,^^16^, ^17^, ^18^^,^^24^^,^^25^^,^^27^^,^^29^^,^^30^^,^^33^, ^34^, ^35^^,^^43^^,^^47^^,^^52^^,^^53^ being most common (59.5%, n = 132/222), followed by unspecified traumatic injuries^1^^,^^7^^,^^16^^,^^25^^,^^29^ (21.6%, n = 48/222), direct blows/falls^11^^,^^12^^,^^14^, ^15^, ^16^^,^^18^, ^19^, ^20^^,^^25^^,^^27^^,^^29^^,^^38^^,^^39^^,^^41^^,^^42^^,^^53^ (11.7%, n = 26/222), and atraumatic injuries^11^^,^^16^^,^^36^ (2.3%, n = 5/222) (Table I).

Sport-specific activity at the time of injury was reported in 214 athletes across 29 studies. Weightlifting was the most frequent activity, reported in 40.7% (n = 87/214) of patients,^1^^,^^5^^,^^7^^,^^10^^,^^15^^,^^20^^,^^25^^,^^27^^,^^30^^,^^34^, ^35^, ^36^^,^^38^^,^^42^^,^^43^^,^^47^^,^^52^ followed by American football^1^^,^^13^^,^^24^^,^^29^^,^^33^ in 26.6% (n = 57/214). Other athletic activities included running (n = 23),^1^^,^^14^ golf (n = 20),^1^^,^^18^^,^^53^ and baseball (n = 16)^1^ (Table I). No specific sporting activity was reported in 9 patient athletes across 2 studies.^18^^,^^25^ Professional athletes comprised 24.8% (n = 53/214) of patients,^12^^,^^13^^,^^17^^,^^29^^,^^35^^,^^36^^,^^42^ while the remainder were either amateur athletes or athletes of an unspecified competitive level (Table I).

The use of diagnostic imaging was reported in 54.2% (n = 150/277) of patients, with magnetic resonance imaging^5^^,^^7^^,^^10^^,^^11^^,^^14^^,^^19^^,^^20^^,^^24^^,^^25^^,^^27^^,^^29^^,^^30^^,^^34^, ^35^, ^36^^,^^38^^,^^41^^,^^43^^,^^47^^,^^52^^,^^53^ accounting for the most common imaging modality (79.3%, n = 119/150), followed by standard radiographs^5^^,^^7^^,^^12^^,^^14^, ^15^, ^16^^,^^19^^,^^20^^,^^24^^,^^25^^,^^27^^,^^30^^,^^33^^,^^34^^,^^36^^,^^38^^,^^39^^,^^41^, ^42^, ^43^^,^^47^^,^^52^^,^^53^ (29.3%, n = 44/150), ultrasound (12.0%, n = 18/150),^10^^,^^12^^,^^24^^,^^25^^,^^27^^,^^30^^,^^34^^,^^36^^,^^38^ and computed tomography (2.7%, n = 4/150).^25^^,^^33^ Pertinent physical examination findings were reported in 54.2% (n = 150/277) of patients, consisting of pain and weakness with triceps extension^7^^,^^10^^,^^12^^,^^14^^,^^17^^,^^19^^,^^20^^,^^24^^,^^27^^,^^29^^,^^30^^,^^33^, ^34^, ^35^, ^36^^,^^38^^,^^41^, ^42^, ^43^^,^^47^^,^^52^^,^^53^ (28.7%, n = 43/150) followed by restricted elbow motion^7^^,^^10^^,^^12^^,^^14^^,^^17^^,^^19^^,^^20^^,^^27^^,^^29^^,^^30^^,^^34^, ^35^, ^36^^,^^39^^,^^41^^,^^43^^,^^47^^,^^53^ (25.3%, n = 38/150), prodromal discomfort along the posterior elbow^5^^,^^10^^,^^27^^,^^29^^,^^38^ (18.7%, n = 28/150), and a palpable gap along the posterior arm (16.0%, n = 24/150).^14^^,^^15^^,^^17^^,^^20^^,^^24^^,^^29^^,^^30^^,^^34^^,^^35^^,^^39^^,^^42^^,^^47^^,^^52^^,^^53^ Anabolic steroid^7^^,^^11^^,^^25^^,^^35^^,^^36^^,^^38^^,^^43^ use was reported in 9 patients, while 8 patients had undergone prior corticosteroid injection^27^^,^^29^^,^^52^ to the posterior elbow.

The presence or absence of concomitant injuries was reported in 51.6% (n = 143/277) of patients, with concomitant injuries present in 12.6% (n = 18/143) of patients across 5 studies.^5^^,^^11^^,^^24^^,^^25^^,^^47^ The most frequently reported concomitant injury was to the ulnar collateral ligament^5^^,^^11^^,^^24^ (n = 8), followed by flexor–pronator injury (n = 7)^5^^,^^11^ and radial head fracture (n = 4)^5^^,^^25^ (Table I).

### Surgical characteristics and techniques

Repair for DTTR was reported at a weighted mean of 2.3 months (range, 0.1-12 months) following injury. Repair technique was reported in 77.3% (n = 214/277) of cases, most commonly involving open transosseous bone tunnel^1^^,^^11^, ^12^, ^13^, ^14^, ^15^, ^16^, ^17^, ^18^^,^^24^^,^^25^^,^^29^^,^^30^^,^^33^^,^^34^^,^^36^^,^^38^^,^^39^^,^^42^^,^^43^^,^^47^^,^^52^ (45.3%, n = 97/214), followed by primary open suture repair^1^^,^^5^^,^^7^^,^^27^ (40.2%, n = 86/214). Additional techniques included open suture anchor^1^^,^^11^^,^^19^^,^^27^^,^^35^^,^^41^^,^^53^ repair (14.0%, n = 30/214), DTTR reconstruction^5^^,^^12^^,^^17^^,^^20^^,^^35^^,^^38^^,^^52^ (3.3%, n = 7/214), and arthroscopic repair^34^ (0.5%, n = 1/214; Table I). Location of fixation consisted of tendon-to-bone repair,^1^^,^^11^^,^^12^^,^^14^, ^15^, ^16^, ^17^, ^18^, ^19^, ^20^^,^^24^^,^^25^^,^^27^^,^^29^^,^^30^^,^^33^, ^34^, ^35^, ^36^^,^^38^^,^^39^^,^^41^, ^42^, ^43^^,^^47^^,^^52^^,^^53^ reported in 80.5% (n = 128/159) of cases, and tendon-tendon repairs^27^ in 2.8% (n = 6/159).

### Postoperative complications and reoperations

The presence or absence of post-operative complications and reoperations was reported for 74.4% (n = 206/277) of patients. A total of 10.2% (n = 21/206) of cases had a reported complication,^5^^,^^11^^,^^18^^,^^25^^,^^29^^,^^38^ most commonly involving tendon rerupture^5^^,^^11^^,^^29^^,^^38^ in 4.4% (n = 9/206) of patients, prolonged pain/weakness^5^^,^^29^^,^^38^ in 2.4% (n = 5/206), and nerve palsy^5^^,^^18^^,^^25^ (1.5%, n = 3/206) involving the ulnar nerve (n = 2) and posterior interosseous nerve (n = 1). Delayed wound healing,^5^ infection,^18^ nerve entrapment,^25^ and a blistering skin rash^5^ were each reported in a single patient. Reoperations were performed in 7.8% (n = 16/206) of patients,^1^^,^^5^^,^^11^^,^^25^^,^^29^^,^^38^ consisting of revision repair^5^^,^^11^^,^^29^^,^^38^ (n = 5), nerve decompression^1^^,^^25^ (n = 4), hardware removal^1^ (n = 2), and ulnar nerve repair^1^ (n = 2). Débridement,^1^ ligament repair,^1^ postrepair reconstruction,^5^ and scar tissue excision^1^ were each performed in a single patient (Table I).

### Return to sport and functional outcomes

RTS was reported in 97.8% (n = 271/277) of patients across all 31 studies, with 93.0% (n = 252/277) successfully returning to sport at a weighted mean of 5.6 months (range, 1.6-10.6 months). A total of 63.6% (n = 42/66) of patients returned to their previous or a higher level of competition,^1^^,^^7^^,^^14^, ^15^, ^16^, ^17^^,^^24^^,^^25^^,^^27^^,^^30^^,^^35^^,^^36^^,^^38^^,^^39^^,^^41^^,^^43^^,^^52^^,^^53^ while 36% (n = 24/66) returned to sport at a lower level.^1^^,^^16^^,^^27^

Individual sporting activities were classified according to the relative injury risk categories defined by Rice,^40^ including contact and limited-contact sports. When reported, athletes participating in contact sports^12^, ^13^, ^14^^,^^17^^,^^19^^,^^24^^,^^29^^,^^33^^,^^41^ achieved successful RTS in 98.2% (n = 54/55) of cases at a weighted mean of 5.3 months (range, 1.6-10.6). For those participating in limited-contact sports,^5^^,^^7^^,^^10^^,^^15^^,^^20^^,^^27^^,^^30^^,^^34^, ^35^, ^36^^,^^38^^,^^39^^,^^42^^,^^43^^,^^47^^,^^52^ successful RTS was reported for 94.4% (n = 68/72) of athletes at a weighted mean of 4.4 months.

Post-operative patient-reported outcome measures were reported at final follow-up (weighted mean 23.4 months; range, 12-108 months). The weighted mean post-operative visual analog s-cale pain score^1^^,^^16^^,^^18^ of 1.7 (n = 97), while the weighted mean Disabilities of the Arm, Shoulder, and Hand (DASH)^11^^,^^17^^,^^18^^,^^38^^,^^39^ score was 4.1 (n = 24). The weighted mean QuickDASH^1^^,^^34^^,^^53^ score was 8.1 (n = 71), and the weighted mean Mayo Elbow Performance Score^1^^,^^11^^,^^18^^,^^34^^,^^36^^,^^39^^,^^43^^,^^53^ was 89.1 (n = 119) (Table I).

## Discussion

Of the 31 identified studies comprising 277 athletes undergoing DTTR repair, males represented 96.4% of patients, with injuries most commonly reported during weightlifting and American football. DTTR repair was frequently performed using a transosseous technique at a weighted mean of 2.3 months following injury. Complications were reported in 10.2% of cases, with tendon rerupture occurring in 4.4%. Successful RTS was reported in 93% of athletes at a mean of 5.2 months following repair, with 63.6% returning to their prior or a higher level of competition.

Weightlifting and American football represented the most common athletic activities resulting in DTTR, accounting for 67.3% of reported cases when athletic activity was reported. Injury typically occurs secondary to eccentric forces applied across the elbow as a result of triceps muscle lengthening during elbow extension,^11^^,^^23^^,^^55^ accompanied by associated muscle contraction during resistance training, primarily occurring during the lowering phase of bench press or overhead pressing movements.^10^^,^^11^^,^^30^^,^^35^^,^^43^^,^^47^ When combined with high training volume, muscle fatigue, or pre-existing tendon degeneration secondary to tendinopathy, the risk of injury to the distal triceps tendon increases during resistance training.^2^^,^^49^ Moreover, prior corticosteroid injection and the use of anabolic steroids have also been implicated in contributing to triceps injury in weightlifting athletes.^23^^,^^28^^,^^44^^,^^45^ Meanwhile, triceps injuries in American football players are frequently associated with axial loading onto an extended upper extremity, commonly associated with blocking and tackling maneuvers. These mechanisms subject the distal triceps to eccentric overload, compounded by repetitive direct contact forces. As such, athletes participating in frequent resistance training, as well as football athletes engaged in repeated blocking, should be advised regarding the risk of DTTR, particularly athletes with a prior diagnosis of triceps tendinitis, and especially athletes utilizing anabolic steroids.

Fixation of distal triceps tears most commonly involved the use of transosseous bone tunnels. In a cadaveric study comparing 10 matched elbows undergoing either transosseous bone tunnel repair or knotless, double-row, anatomic footprint, suture anchor repair, Carpenter et al^8^ reported no significant difference in tendon displacement during cyclic loading or load-to-failure testing. In a multicenter retrospective cohort of 56 cases, Horneff et al^22^ reported excellent post-operative Mayo Elbow Performance Scores using both transosseous and suture anchor techniques (92.8 vs. 95.6; *P* = .25, respectively). While DASH scores following transosseous repair were 3 points lower (*P* = .03), this result was reported as not being clinically meaningful.^22^ Post-operative visual analog scale scores were equivalent between techniques (*P* = .6), while 2 tendon reruptures occurred in each group.^21^ In a systematic review comprising 16 studies (n = 591 patients), suture anchor repair was reported to yield superior results to transosseous repair based on isokinetic strength testing (95% vs. 82%), complication rates (8% vs. 18%, *P* = .008), and rerupture rates (2% vs. 7%, *P* = .03), respectively.^48^ Future prospective studies comparing fixation constructs with standardized RTS definitions are warranted to more accurately assess long-term outcomes in athletes returning to high-demand activities.

While overall complication rates following DTTR were relatively low, the tendon rerupture was 4.4%. Brush et al^6^ reported rerupture rates following transosseous tunnel repair, suture anchor repair, and combined transosseous tunnel plus suture anchor repair of 6.1%, 4.4%, and 12.5%, respectively (*P* = .260). Contributing factors reported to play a role in tendon rerupture include a premature RTS,^5^^,^^16^ inadequate biological healing at the tendon–bone interface,^41^ and overly aggressive rehabilitation.^5^^,^^9^ While anabolic and corticosteroid use are established risk factors for DTTR,^28^^,^^45^ their continued use is reported to contribute to repair failure by weakening tendon structure. Specifically, Lee et al^26^ reported that prior recent anabolic steroid use led to a 3.4-fold increase in the 1-year revision rate following DTTR (*P* < .001), with additional studies citing steroid use as a modifiable risk factor during surgical planning and post-operative management.^9^^,^^22^ While both acute and chronic DTTR undergoing primary repair generally yield satisfactory functional outcomes,^21^^,^^31^ there is no clear evidence that delayed treatment alone predicts failure.^50^^,^^54^ Further high-level evidence studies are warranted to clarify the impact of surgical timing on long-term outcomes and more accurately identify specific risk factors leading to distal tricep tendon rerupture.

A major limitation of this review was the predominance of small case series and case reports with lower levels of evidence due to selection bias, lack of control groups, and incomplete reporting of outcomes. As such, there is a need for higher-quality, prospective investigation to better guide management in athletes based on competition level, specific sport, and particularly those reporting preinjury anabolic steroid use. Moreover, the generalizability of these findings to broader athletic populations may be limited as differentiation between amateur and professional athletes was infrequent, preventing analysis evaluating differences in outcomes or RTS rate/timing based on competition level. Substantial heterogeneity was present among studies based on patient demographics, mechanisms of injury, tear characteristics, surgical techniques, rehabilitation protocols, and outcome measures. Furthermore, the heterogeneous spectrum of pathology, including both partial and full-thickness ruptures, further limited the generalizability of outcomes following surgical repair and/or reconstruction. This variability effectively prevented the performance of any meaningful statistical analysis examining these factors based on sport or level of competition. Detailed reporting of injury mechanisms, rehabilitation progression, and patient comorbidities was inconsistent, further limiting the ability to conclude specific risk factors for initial injury or rerupture. Finally, we only reported on surgical repair of these injuries, without any comparison to nonoperative management outcomes, which may be indicated in patients with partial tendon tears with preserved strength and function.

## Conclusion

The vast majority of patients undergoing DTTR repair are male, most commonly engaged in weightlifting or American football. Successful RTS was reported in 93% of patients at a mean of 5.2 months following repair, while tendon rerupture was reported in 4.4% and reoperations in 7.8%.

## Disclaimers:

Funding: No funding was disclosed by the authors.

Conflicts of interest: D.M.K. has received support for education from Synthes, Smith & Nephew, Elite Orthopedics, and Medwest Associates; hospitality payments from Arthrex, Elite Orthopaedics, Encore Medical, Stryker, and Smith & Nephew; honoraria from Encore Medical; and a grant from Arthrex. M.V.S. has received speaking and faculty, education, and hospitality payments from Arthrex; and education and hospitality payments from Elite Orthopaedics, and hospitality payments from Medical Device Business Services. R.H.B. has received support for education and hospitality payments from Elite Orthopaedics and hospitality payments from Zimmer Biomet. The other authors, their immediate families, and any research foundations with which they are affiliated have not received any financial payments or other benefits from any commercial entity related to the subject of this article.
