# Supplementary material for: Distal triceps tendon rupture repair results in high return to sport rates for amateur and professional athletes: a systematic review
Source: JSES Rev Rep Tech. 2026 Feb 6;6(2):100694. doi: 10.1016/j.xrrt.2026.100694 (PMC12989953; doi:10.1016/j.xrrt.2026.100694)
Supplement: Appendix Table 1 [file mmc1.docx]

**Appendix Table 1. Joanna Briggs Institute (JBI) critical appraisal tool for case series**

| **Study** | **Q1** | **Q2** | **Q3** | **Q4** | **Q5** | **Q6** | **Q7** | **Q8** | **Q9** | **Q10** | **Total Percentage** |
| --- | --- | --- | --- | --- | --- | --- | --- | --- | --- | --- | --- |
| Agarwalla (2023)[^1^](#_ENREF_1) | Y | Y | Y | Y | Y | Y | Y | Y | N | Y | 90% |
| Balazs (2016)[^5^](#_ENREF_5) | Y | Y | Y | Y | Y | Y | Y | Y | Y | Y | 100% |
| Dunn (2019)[^11^](#_ENREF_11) | Y | Y | Y | Y | Y | Y | Y | Y | N | Y | 90% |
| Finstein (2015)[^13^](#_ENREF_13) | Y | Y | Y | Y | Y | Y | N | N | Y | Y | 80% |
| Gruber (2024)[^16^](#_ENREF_16) | Y | Y | Y | Y | N | Y | U | Y | N | Y | 70% |
| Hall (2021)[^18^](#_ENREF_18) | Y | Y | Y | Y | N | Y | Y | Y | N | Y | 80% |
| Kose (2015)[^25^](#_ENREF_25) | Y | Y | Y | Y | Y | Y | Y | Y | N | Y | 90 |
| Lempainen (2011)[^27^](#_ENREF_27) | Y | Y | Y | Y | Y | Y | Y | Y | N | Y | 90% |
| Mair (2004)[^29^](#_ENREF_29) | Y | Y | Y | Y | Y | Y | Y | Y | N | Y | 90 |
| Total Percentage | 100% | 100% | 100% | 100% | 78% | 100% | 78% | 89% | 22% | 100% | 87% |

Legend: Q, question; Y, yes; N, no; U, unclear; NA, not applicable

Q1, Were there clear criteria for inclusion in the case series?

Q2, Was the condition measured in a standard, reliable way for all participants included in the case series?

Q3, Were valid methods used for identification of the condition for all participants included in the case series?

Q4, Did the case series have consecutive inclusion of participants?

Q5, Did the case series have complete inclusion of participants?

Q6, Was there clear reporting of the demographics of the participants included in the study?

Q7, Was there clear reporting of clinical information of the participants?

Q8, Were the outcomes or follow-up results of cases clearly reported?

Q9, Was there clear reporting of the presenting sites’/clinics’ demographic information?

Q10, Was statistical analysis appropriate?
