# Supplementary material for: Distal triceps tendon rupture repair results in high return to sport rates for amateur and professional athletes: a systematic review
Source: JSES Rev Rep Tech. 2026 Feb 6;6(2):100694. doi: 10.1016/j.xrrt.2026.100694 (PMC12989953; doi:10.1016/j.xrrt.2026.100694)
Supplement: Appendix Table 2 [file mmc2.docx]

**Appendix Table 2. Joanna Briggs Institute (JBI) critical appraisal tool for case reports**

| **Study** | **Q1** | **Q2** | **Q3** | **Q4** | **Q5** | **Q6** | **Q7** | **Q8** | **Total Percentage** |
| --- | --- | --- | --- | --- | --- | --- | --- | --- | --- |
| Bunshah (2015)[^7^](#_ENREF_7) | Y | Y | Y | Y | Y | Y | Y | Y | 100% |
| Chorba (2024)[^10^](#_ENREF_10) | Y | Y | Y | Y | Y | Y | U | Y | 88% |
| Figueiredo (2019)[^12^](#_ENREF_12) | Y | Y | Y | Y | Y | Y | U | Y | 88% |
| Goodrich (2018)[^14^](#_ENREF_14) | Y | Y | Y | Y | Y | Y | Y | Y | 100% |
| Greer (2005)[^15^](#_ENREF_15) | Y | Y | Y | Y | Y | Y | Y | Y | 100% |
| Gupta (2017)[^17^](#_ENREF_17) | Y | Y | Y | Y | Y | Y | U | Y | 88% |
| Hernandez (2024)[^19^](#_ENREF_19) | Y | Y | Y | Y | Y | Y | Y | Y | 100% |
| Holmes (2020)[^20^](#_ENREF_20) | Y | Y | Y | Y | Y | Y | Y | Y | 100% |
| Khalil (2018)[^24^](#_ENREF_24) | Y | Y | Y | Y | Y | Y | Y | Y | 100% |
| Mangano (2015)[^30^](#_ENREF_30) | Y | Y | Y | Y | Y | Y | Y | Y | 100% |
| Naito (2013)[^33^](#_ENREF_33) | N | U | Y | Y | Y | Y | Y | Y | 75% |
| Naula (2022)[^34^](#_ENREF_34) | Y | Y | Y | Y | Y | Y | Y | Y | 100% |
| Nikolaidou (2014)[^35^](#_ENREF_35) | Y | Y | Y | Y | Y | Y | Y | Y | 100% |
| Ntourantonis (2023)[^36^](#_ENREF_36) | Y | Y | Y | Y | Y | Y | Y | Y | 100% |
| Pilih (2022)[^38^](#_ENREF_38) | Y | Y | Y | Y | Y | Y | Y | Y | 100% |
| Qin (2021)[^39^](#_ENREF_39) | Y | Y | Y | Y | Y | Y | U | Y | 88% |
| Schreiderer (2017)[^41^](#_ENREF_41) | N | Y | Y | Y | Y | Y | Y | Y | 88% |
| Sherman (1984)[^42^](#_ENREF_42) | Y | Y | Y | N | N | Y | N | Y | 63% |
| Shivdasani (2024)[^43^](#_ENREF_43) | Y | Y | Y | Y | Y | Y | Y | Y | 100% |
| Tramer (2021)[^47^](#_ENREF_47) | Y | Y | Y | Y | Y | Y | Y | Y | 100% |
| Weistroffer (2003)[^52^](#_ENREF_52) | Y | Y | Y | Y | Y | Y | U | Y | 88% |
| Welborn (2023)[^53^](#_ENREF_53) | N | Y | Y | Y | Y | Y | Y | Y | 87.5 |
| Total Percentage | 83% | 91% | 96% | 91% | 91% | 96% | 70% | 96% | 89% |

Legend: Q, question; Y, yes; N, no; U, unclear; N/A, not applicable

Q1, Were patient’s demographic characteristics clearly described?

Q2, Was the patient’s history clearly described and presented as a timeline?

Q3, Was the current clinical condition of the patient on presentation clearly described?

Q4, Were diagnostic tests or assessment methods and the results clearly described?

Q5, Was the intervention(s) or treatment procedure(s) clearly described?

Q6, Was the post-intervention clinical condition clearly described?

Q7, Were adverse events (harms) or unanticipated events identified and described?
